# AI-based analysis algorithm incorporating nanoscale structural variations and measurement-angle misalignment in spectroscopic ellipsometry

**DOI:** 10.1515/nanoph-2025-0515

**Published:** 2025-12-09

**Authors:** Juwon Jung, Leeju Hwang, Nagyeong Kim, Kibaek Kim, Seri Kim, Jongkyoon Park, Won Chegal, Yong Jai Cho, Young-Joo Kim

**Affiliations:** Department of Mechanical Engineering, 26721 Yonsei University, Seoul, Republic of Korea; Semiconductor and Display Metrology Group, Korea Research Institute of Standards and Science, 267 Gajeongno, Yuseong-Gu, Daejeon, 34113, Republic of Korea; Graduate School of Analytical Science and Technology (GRAST), Chungnam National University, Daejeon, 34134, Republic of Korea

**Keywords:** spectroscopic ellipsometry, fabrication-induced structural variation, measurement-angle, nanostructure

## Abstract

Spectroscopic ellipsometry (SE) is a powerful, non-destructive technique for nanoscale structural characterization. However, conventional SE data analysis typically assumes perfectly periodic specimen structures, overlooking fabrication-induced structural variations and thereby reducing the accuracy of predicted structural parameters. We have developed an enhanced analysis framework that explicitly accounts for both nanoscale structural variations and measurement-angle misalignment by introducing the concept of an average Mueller matrix (MM), which represents statistical distributions of nanoscale structures. In addition, we introduce a high-throughput MM-generation neural network that enables rapid data preparation by approximating rigorous coupled-wave analysis (RCWA) simulations for large numbers of specimens across a broad range of structural parameters. The model achieves a mean-squared error of 9.99 × 10^−8^ MSE when validated against RCWA-simulated MM data for one-dimensional SiO_2_ nanogratings. Finally, we apply our analysis framework to experimentally measured MM data, achieving highly accurate dimensional predictions with errors below 0.4 nm when compared with structural parameters measured by scanning electron microscopy (SEM). We believe that this analysis algorithm significantly advances the potential for high-precision SE-based metrology in semiconductor, photonic, and display manufacturing.

## 1 Introduction

Spectroscopic ellipsometry (SE) is a non-destructive technique widely used for precise characterization of physical and optical properties, such as thickness and refractive index [1], [2], as well as nanoscale structural geometries of thin films and nanostructures [3], [4]. SE finds broad applications in industries including semiconductors [5], [6], displays [7], and advanced materials [8], [9]. Determining structural parameters from SE-measured optical responses, such as the Mueller matrix (MM), constitutes an inverse problem [10]. Solving this inverse problem using conventional simulation-based least-squares fitting methods [11], [12], [13], [14], [15] often requires extensive computations and is time-consuming [16], [17]. To address these limitations, artificial neural networks (ANNs) have recently been investigated to accelerate SE data analysis [4], [18], [19].

Our research group previously developed a neural network–assisted two-step algorithm for analyzing Mueller matrix spectroscopic ellipsometry (MMSE) data of one-dimensional (1D) nanograting structures [20]. In the first step, a library of rigorous coupled-wave analysis (RCWA) simulations was searched to identify the parameter node whose MM best matched the measured MM. The second step refined this initial estimate using MMs from neighboring nodes to improve accuracy. More recently, we extended this framework by incorporating optical constant prediction and replacing the library search with a neural network, yielding a fully neural network–based analysis method with both faster speed and higher accuracy [21].

Despite these advances, most existing analysis approaches, including ANN and simulation-based methods, assume perfectly periodic nanostructures [14], [18], [22], [23]. In reality, the probing beam interacts with numerous nanostructures, and the measured optical response represents an average over structurally non-uniform regions [24]. This idealization neglects fabrication-induced variations in key geometrical parameters – such as height, period, and linewidth – that strongly influence the optical response [25], [26], [27]. As a result, interpreting the measured MM as arising from a single uniform structure can compromise the accuracy of structural parameter extraction. Linewidth-related parameters, including top and bottom critical dimensions, are particularly sensitive because they also encode sidewall angles and 3D pattern profiles [28], [29], [30]. Therefore, accurately incorporating fabrication-induced structural variations is essential for reliable parameter analysis and optimal device performance [28], [31], [32].

In addition, although the beam incident angle and azimuthal angle are typically fixed during SE measurements, small misalignments are inevitable in practice. These deviations introduce additional uncertainty and can reduce the robustness of parameter extraction [33], [34], [35], [36].

In this study, we propose a neural network–based structural parameter prediction methodology that explicitly accounts for both fabrication-induced structural variations and measurement-angle misalignment. To achieve this, we constructed a training dataset incorporating both effects. A realistic region of the structural parameter space was first defined to model with fabrication-induced variations. Multiple sampling points were then generated within this region using a strategy that efficiently captures its characteristics while minimizing the number of required points. For each sampled point, the corresponding MM was predicted using a network-based MM-generation neural network, enabling rapid and accurate MM-generation compared with conventional RCWA simulations [37]. The generated MMs were then averaged to produce representative MMs that reflect structural variations. Simultaneously, misalignments in incident and azimuthal angles were parameterized and integrated into the MM-generation and averaging process to account for measurement uncertainty.

The resulting dataset, containing averaged MMs under both structural and angular variations, was used to train our neural network–based two-step algorithm, following the architecture of our previous work [20], [21]. Trained on this variation-inclusive dataset, the algorithm achieved high accuracy and robustness in parameter prediction for both simulated and experimental data. By explicitly modeling real-world structural variations and measurement-angle uncertainty, the proposed framework overcomes idealized assumptions and substantially enhances the precision, reliability, and practical applicability of SE-based nanostructure metrology.

## 2 Methodology

### 2.1 Modeling structural variations and measurement-angle misalignment

MMSE characterizes periodic nanostructures by measuring the polarization changes of light reflected from fabricated samples. These changes are represented by a single MM, which captures the averaged optical response within the illuminated area [14], [38]. In practice, however, fabricated nanostructures often deviate from ideal periodic designs, exhibiting nanometer-scale variations in shape and dimension across periods due to inherent process fluctuations [31], [32]. Such structural variations induce subtle differences in the optical response of each structure, thereby influencing the measured MM.

**Figure 1:**
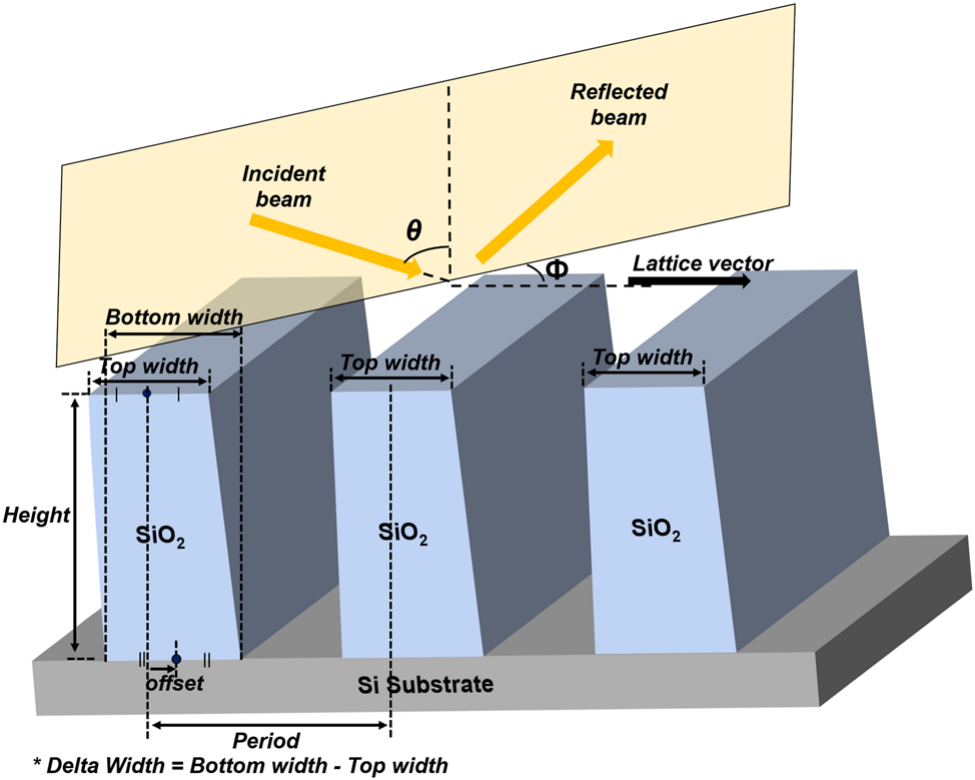
Schematic of the 1D grating structure showing the definitions of the structural parameters and the incident beam.

**Figure 2:**
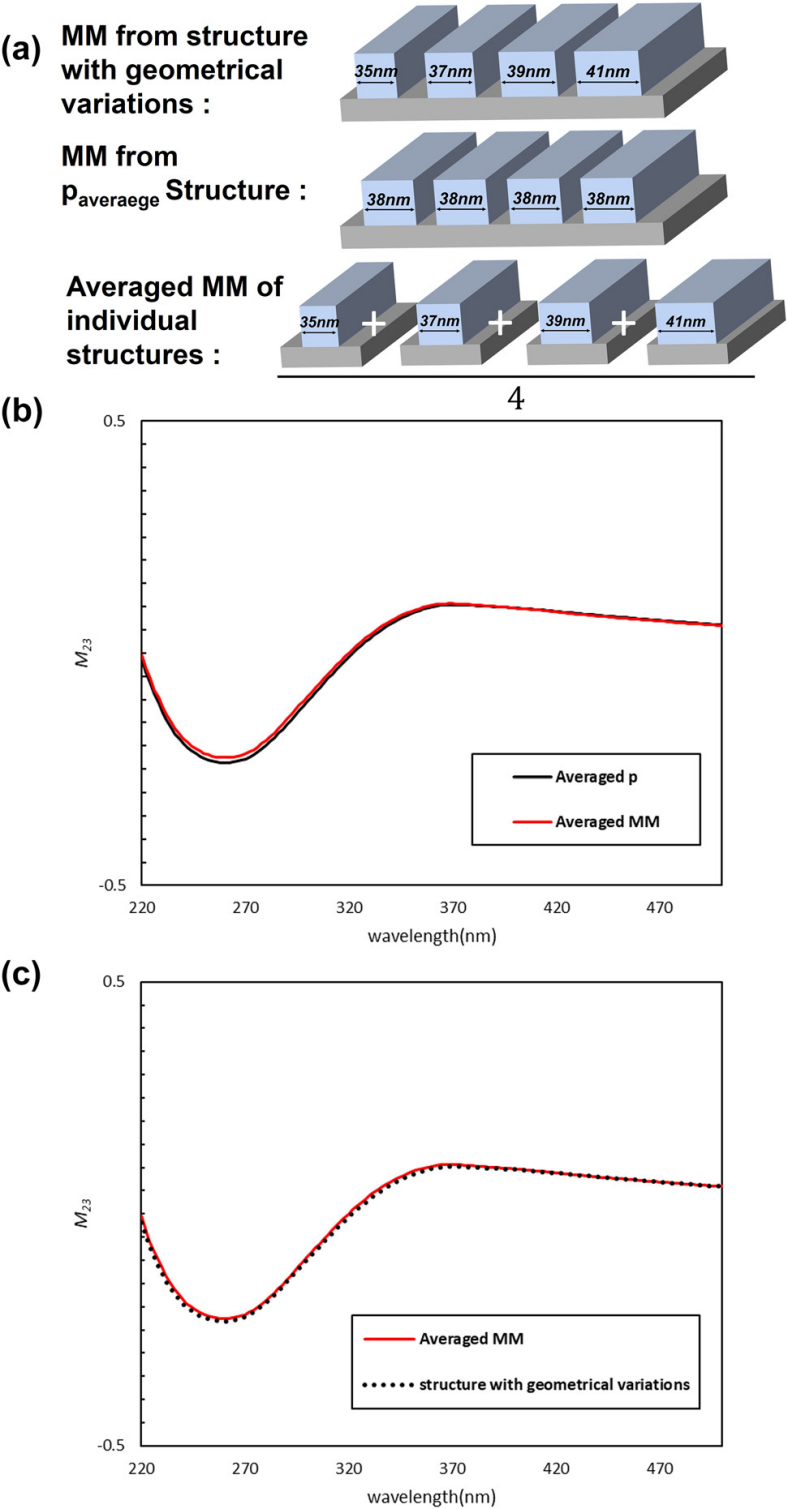
Overall comparison of the *M*_*23*_ element for representative non-uniform structures with geometrical variations: (a) schematic of three representative structures; (b) results obtained using the averaged structural parameter (*p*_average_) and averaged MM approaches; and (c) comparison between the averaged MM and the direct MM calculated using FDTD simulations for non-uniform structures with geometrical variations.

In this work, we consider a one-dimensional SiO_2_ grating, defined by the structural parameters and measurement conditions illustrated in Figure 1. To investigate the effect of structural variations on the MM, we analyzed several representative cases, as shown in Figure 2(a). Specifically, we examined a structure with geometrical variations composed of four different linewidths (35, 37, 39, and 41 nm) arranged sequentially, mimicking a non-uniform specimen measured by SE. For this structure, the MM was first calculated for a grating with the averaged value *p*_average_ = 38 nm, using RCWA simulations. Motivated by the fact that SE measurements reflect the average optical response from multiple structures, we then implemented an MM averaging approach, in which the RCWA-simulated MMs of individual periodic structures (35, 37, 39, and 41 nm) were averaged to produce a representative MM. Figure 2(b) compares the *M*_*23*_ element of the MM obtained from the two approaches. Although both correspond to the average value of 38 nm, noticeable differences are observed, demonstrating that assuming a perfectly periodic structure with an averaged value is insufficient. Accounting for structural variations is therefore crucial for precise nanostructure analysis.

To further validate the MM averaging approach, the same non-uniform specimen was modeled using finite-difference time-domain (FDTD) simulation, which is significantly more computationally demanding. Figure 2(c) shows that the *M*_*23*_ element obtained from the MM averaging approach closely matches the FDTD result. Across 40 different non-uniform specimens, the MM results from the two methods were compared using the mean squared error (MSE), and the averaged MSE of 3.12 × 10^−5^ confirms the high level of agreement (see Supplementary Section 1). These results demonstrate that the proposed MM averaging approach can effectively analyze measured MMs from non-uniform structures with geometrical variations.

Building on this concept, we defined the parameter space of structural variations and generated representative discrete combinations using an efficient sampling strategy. In parallel, a neural-network-based MM generator was developed to rapidly produce MMs for large numbers of structures, enabling efficient large-scale analysis while accurately capturing the effects of structural variations.

#### 2.1.1 Definition of structural parameters and sampling space

We define four structural parameters for the one-dimensional grating shown in Figure 1: *h* (height), *a*_*w*_ (average width, the average of bottom and top widths), *d*_*w*_ (delta width, the difference between bottom and top widths), and *off* (offset, i.e., the lateral displacement of the bottom-width center position relative to the top-width center). The corresponding parameter ranges are *h* ∈ [80, 120] nm, *a*_*w*_ ∈ [30, 45] nm, *d*_*w*_ ∈ [0, 15] nm, and *off* ∈ [−10, 10] nm. In addition, the variation ranges of measurement-angles are defined as incident angle (*θ* ∈ [68°, 72°]) and azimuthal angle (*ϕ* ∈ [41°, 49°]).

A specific combination of structural parameters is represented by a center vector *p*_*c*_ = [*h*_*c*_, *aw*_*c*_, *dw*_*c*_, *off*_*c*_] and a variation-range parameter vector *p*_*r*_ = [*h*_*r*_, *aw*_*r*_, *dw*_*r*_, *off*_*r*_]. The parameter-space domain Ω is:

$$\Omega =\left[p_{c}-\frac{p_{r}}{2},p_{c}+\frac{p_{r}}{2}\right],$$

which defines a four-dimensional hypervolume. The maximum allowed variation for each variable (the components of *p*_*r*_) was set to 10 nm, consistent with typical fabrication tolerances in state-of-the-art semiconductor processes [15], [39]. Sampling is performed within this domain Ω.

#### 2.1.2 Random symmetric sampling

**Figure 3:**
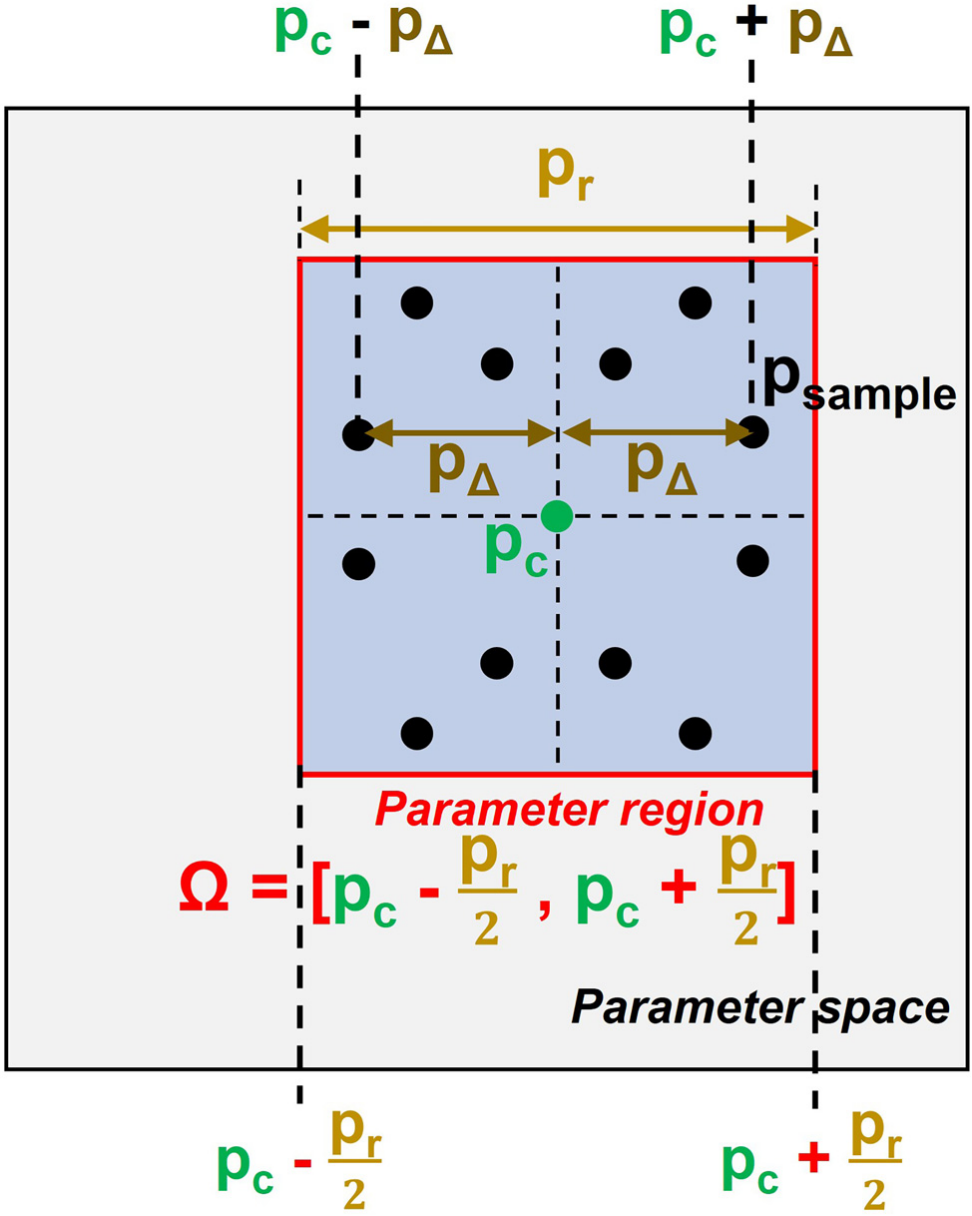
Schematic illustration of the data-generation process accounting for structural variations.

In this study, we propose an efficient high-dimensional sampling strategy termed Random Symmetric Sampling. Within the parameter domain Ω, a local displacement vector *p*_Δ_ is generated relative to *p*_*c*_, where each component corresponding to a structural parameter is randomly sampled from the interval [0, *p*_*r*_/2], as illustrated in Figure 3. Since *p*_Δ_ is four-dimensional, independently assigning positive or negative signs to its components generates 2^4^ = 16 symmetric points. By generating *n* such local displacement vectors and applying this symmetric expansion, a total of *N* = 16n sampling points is obtained. These points are then used as inputs for the MM-generation process.

To ensure uniform coverage across the parameter space, the number of sampling points per unit hypervolume was fixed. Specifically, the sampling density was set to 96 points per (1 nm)^4^, calculated based on the total parameter-space volume and applied uniformly within each *p*-region (see Supplementary Section 2 for details). Consequently, the total number of samples scales proportionally with the four-dimensional parameter-space volume, maintaining representativeness across all ranges. This approach ensures that the sampling density remains consistent, rather than allocating an equal number of points to both small (e.g., 1 nm) and large (e.g., 10 nm) variation ranges. The sampling procedure within a defined parameter region is illustrated in Figure 3. For intuitive understanding, a simplified two-dimensional parameter space is shown under a specific measurement-angle. In this example, local displacements are generated, symmetrically extended, and uniformly distributed within the sampling region. Random symmetric sampling thus combines local randomness with symmetric extension, providing efficient coverage of the parameter space while substantially reducing computational cost compared with exhaustive grid sampling.

#### 2.1.3 Parameterization of measurement-angles

During SE measurements, the incident angle (*θ*) and azimuthal angle (*ϕ*) are typically fixed. However, small but uncontrollable misalignments may occur due to mechanical or environmental fluctuations. Because the measurement angles strongly influence the MM response, neglecting them can introduce significant errors in structural parameter extraction. To account for these effects, we define a measurement-angle vector *A* = [*θ*, *ϕ*] as a variable parameter, with a corresponding domain *A*. The complete parameter space is thus expressed as Ω × *A*, where Ω represents the structural parameter domain. Sampling points within this extended space are denoted as (*p*, *A*) ∈ Ω × *A*, and the corresponding MM is written as *MM* (*p*, *A*). In this study, *A* was explicitly sampled alongside *p*, and the resulting values were incorporated into MM simulations. This parameterization allows for realistic modeling of both structural variations and measurement-angle uncertainty, enhancing the robustness and accuracy of subsequent structural analysis.

### 2.2 *M*_avg_ generation through the development of MM generation neural networks (MMgen)

In Section 2.1, sampling points representing different parameter combinations were generated within the defined parameter space. Obtaining the corresponding MM for each point typically requires RCWA or other electromagnetic simulations. However, performing thousands of such simulations is computationally intensive, creating a bottleneck for real-time implementation and large-scale training data generation.

**Figure 4:**
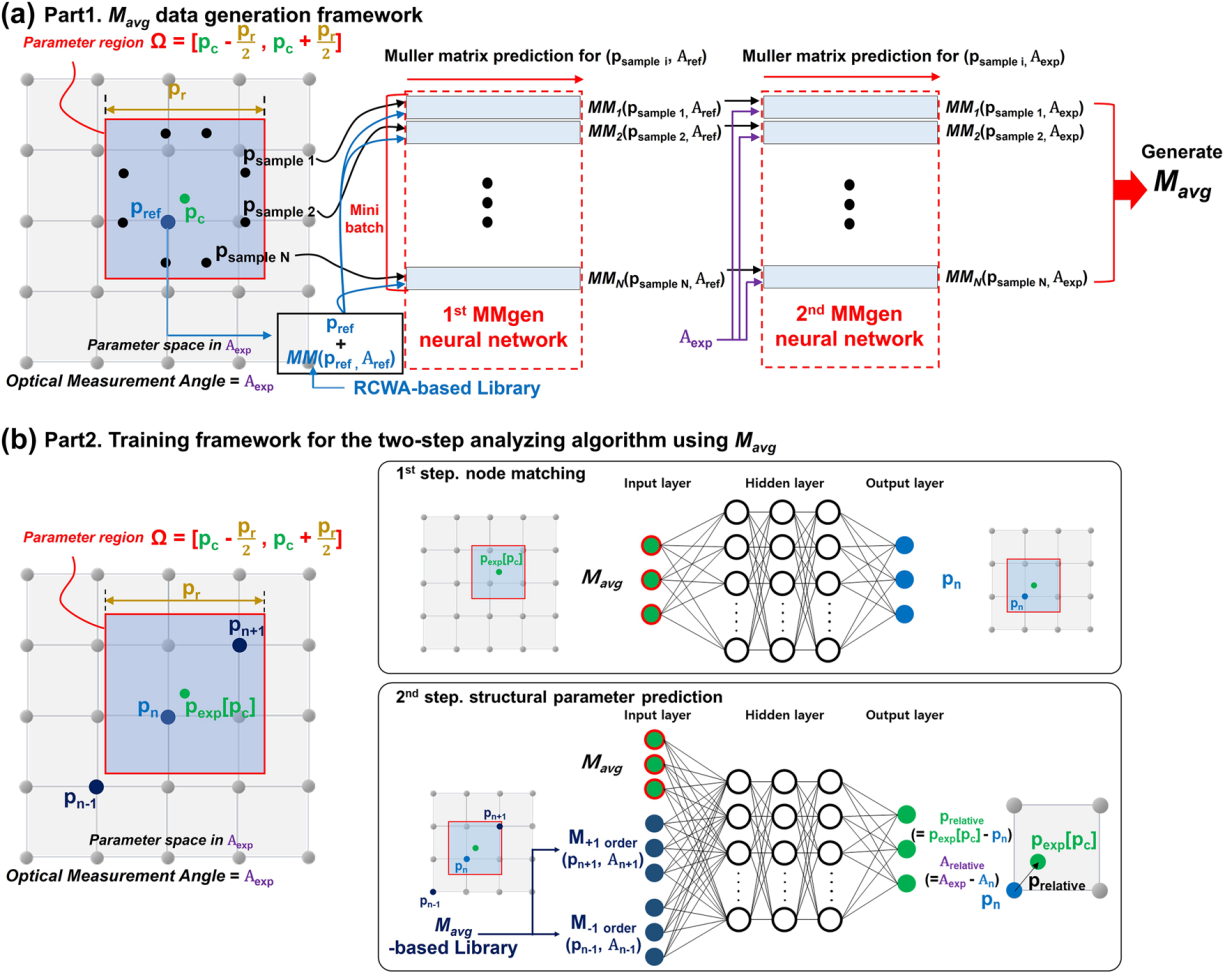
Overall framework of the enhanced two-step analyzing algorithm for structural parameter prediction accounting for specimen-induced structural variations and measurement-angle misalignment: (a) Part 1: *M*_avg_ data-generation framework using MMgen neural networks; (b) Part 2: Training framework for the two-step analyzing algorithm using *M*_avg_.

As an alternative, Jiang et al. [4] proposed a neural network–based model that learns the relationship between structural parameters and reflection ellipsometry measurements (Ψ, Δ), replacing RCWA simulations. Building on this approach, we introduce a high-speed, neural network–based MM-generation framework, termed MMgen, which directly generates the MM corresponding to both structural and measurement-angles parameters. MMgen consists of two sequential neural networks: the first generates the MM with reference to structural parameters, and the second corrects the MM for specific measurement-angle conditions. The architecture is illustrated in Figure 4(a).

The first neural network generates the MM corresponding to a given structural parameter. A reference library, precomputed using RCWA simulations at 5 nm intervals, is used to locate the closest lattice point and its corresponding MM. The network input includes the reference parameter vector (*p*_ref_), the difference (*p*_sample_ − *p*_ref_), and the reference MM, *MM* (*p*_ref_, *A*_ref_), obtained from the RCWA library. The output is the MM, *MM* (*p*_sample_, *A*_ref_), corresponding to the actual structural parameter *p*_sample_ evaluated at fixed reference measurement-angles *A*_ref_ (*θ* = 70°, *ϕ* = 45°). The second neural network refines this result by correcting for the actual measurement-angle conditions. It takes as input the MM generated by the first network, *MM* (*p*_sample_, *A*_ref_), together with *p*_sample_ and the experimental angles *A*_exp_. The output is the corrected MM, *MM* (*p*_sample_, *A*_exp_), corresponding to the full parameter set (*p*_sample_ and *A*_exp_). Using MMgen, MMs for arbitrary combinations of *p*_sample_ and *A*_exp_ can be generated with high speed while maintaining RCWA-level accuracy. This efficiency is further enhanced through GPU-based parallelization, ensuring that computational cost does not scale linearly with the number of samples. As a result, MMgen is highly effective for computing *M*_avg_ across numerous samples reflecting structural variations. The MMs generated by MMgen for each sampling point are averaged according to the structural distribution:

$$M_{\text{avg}}=\frac{\int_{\Omega }f\left(p\right)\cdot M\left(p\right)dp}{\int_{\Omega }f\left(p\right)dp},$$

where *M* (*p*) denotes the predicted MM for a given structural parameter *p*, and *f* (*p*) is the probability density function describing the structural distribution. In this study, we assume that all structures occur with equal probability, i.e., *f* (*p*) = const. Under this assumption, the average MM can be simplified to:

$$M_{\text{avg}}\approx \frac{1}{N}\overset{N}{\underset{i=1}{\sum }}M\left(p_{\text{sample,}i}\right),$$

where *N* is the number of sample points accounting for structural variations. The resulting *M*_avg_ provides a representative MM that approximates the optical response of structures with geometrical variations within the illuminated region. Based on this framework, parameter combinations (*p*, *A*) are sampled within the extended space (Ω × *A*) (Section 2.1). MMgen generates the MM for a large number of structures at each sampling point, which are then averaged to obtain *M*_avg_. This integrated procedure ensures that the entire pipeline – from MM-generation at sampling points to *M*_avg_ computation – proceeds as a streamlined process, enabling rapid and accurate generation of extensive datasets. The resulting *M*_avg_ values provide essential input for subsequent algorithm development and structural parameter analysis.

### 2.3 Enhanced two-step analyzing algorithm accounting for structural variations and measurement-angles misalignment

Based on the training dataset generated in the preceding steps, we developed an enhanced two-step analyzing algorithm that simultaneously predicts structural parameters and measurement-angle conditions while accounting for fabrication-induced structural variations. This framework extends our previously proposed two-step algorithm [21] and can flexibly adjust the output layer to match the number of parameters to be predicted. The networks are trained in a supervised manner using simulated MMs as inputs and the corresponding structural parameters and measurement angles as labels, enabling accurate parameter inference from MM data. In this study, the architecture is explicitly extended to incorporate both structural variations and measurement-angle uncertainty.

The overall framework consists of two main parts, as illustrated in Figure 4.

**Part 1:** ***M***_**avg**_ **data-generation framework using the MMgen neural** **networks**

In Part 1 (Figure 4(a)), random regions are selected within the structural parameter space (Ω × *A*), and multiple sampling points are generated within each region to represent structural variations. For each sampling point, the MMgen neural networks generate the corresponding MM. Precomputed reference data are incorporated to enhance generation accuracy, while GPU-based parallel mini-batch processing enables efficient computation of large numbers of MMs. By averaging the MMs of all structures in a specific region, the average Mueller matrix *M*_avg_ is obtained, providing an optical response that realistically reflects fabrication-induced structural variations for a given measurement-angles.

**Part 2: Training framework of the two-step analyzing algorithm using** ***M***_**avg**_

In Part 2 (Figure 4(b)), the enhanced two-step algorithm is trained using the *M*_avg_ datasets generated in Part 1. The training dataset is produced by defining the parameter-space domain via *p*_*c*_ and *p*_*r*_ (see Figure 3), sampling the space, and generating the corresponding MMs. The algorithm takes a single *M*_avg_ as input and predicts the center parameter vector *p*_exp_[*p*_*c*_] of the structural distribution along with the measurement-angles *A*_exp_.

In the first step, as shown in the upper part of Figure 4(b), the node-matching algorithm takes the input *M*_*avg*_ and identifies the closest node point. Specifically, it compares the center point of the experimental parameter space, denoted as *p*_exp_[*p*_*c*_], with the predefined node points distributed at 5-nm intervals across the parameter space. The node p_n_ with the shortest Euclidean distance to *p*_exp_[*p*_*c*_] is selected. The center parameter vector of this node *p*_*n*_, along with its associated reference angles, serves as the zeroth-order reference for subsequent refinement.

In the second step, illustrated in the lower part of Figure 4(b), the input *M*_avg_ is combined with the MMs of the surrounding −1 and +1 order nodes. The node *p*_*n*_ predicted by the first network is defined as the zeroth-order node (*p*_*n*_, *A*_*n*_), where *A*_*n*_ = *A*_ref_ corresponds to the reference measurement angles (e.g., *p*_*r*_ = 5 nm, *θ* = 70°, *ϕ* = 45°). Using this reference, the +1 node (*p*_*n*+1_, *A*_*n*+1_) is obtained by incrementing *p*_*c*_ by +5 nm and adopting the maximum range of *p*_*r*_ and measurement angles (e.g., *p*_*r*_ = 10 nm, *θ* = 72°, *ϕ* = 49°). Similarly, the −1 node (*p*_*n*−1_, *A*_*n*−1_) is defined by decrementing *p*_*c*_ by −5 nm and adopting the minimum range of *p*_*r*_ and angles (e.g., *p*_*r*_ = 0 nm, *θ* = 68°, *ϕ* = 41°). The MMs corresponding to these ±1-order nodes are retrieved from the pre-generated *M*_avg_-based library, constructed using the MM-generation framework described in Section 2.2. Together with the measured MM, these three MMs are used as inputs to the second-step neural network, which is trained to predict the relative displacement vector (*p*_relative_, *A*_relative_). This vector represents the offset of the zeroth-order node (*p*_*n*_, *A*_*n*_). The final predicted parameters are reconstructed by adding this relative vector to the reference node, i.e., (*p*_exp_, *A*_exp_) = (*p*_*n*_, *A*_*n*_) + (*p*_relative_, *A*_relative_).

In this manner, the two-step analyzing algorithm, trained using the *M*_*avg*_ dataset, can predict structural parameters directly from measured MM data obtained via spectroscopic ellipsometry. This approach enables accurate estimation of nanostructure parameters even in the presence of fabrication-induced structural variations and measurement-angle misalignment.

## 3 Results and discussion

To evaluate the impact of incorporating structural variations and measurement-angle misalignment, we constructed two cases of parameter variables for the two-step analyzing algorithm.

In the first case, only the four structural parameters were treated as variables, with data based on perfectly periodic structures generated using RCWA simulations and an RCWA-based library. Both datasets were used to train the MMgen neural networks and the two-step analyzing algorithm. Additional details are provided in Sections 3.1 and 3.2.

In the second case, the model incorporates not only the four structural parameters but also the two measurement angles and structural variations. This case uses the *M*_*avg*_ dataset and its corresponding *M*_*avg*_-based library to train the two-step analyzing algorithm, as described in Section 3.3. Further information on each dataset is provided in Supplementary Section 3.

The two-step neural network architecture was identical across datasets in terms of input and hidden layers. In **Step 1**, the input dimension was 320 × 15 × 1 (MM for a single measurement-angle). In **Step 2**, the input dimension was 320 × 15 × 3 (MM for *M*_exp_ and ± 1 nodes). Both Step 1 and Step 2 networks employed a Swin transformer for the hidden layers, which is widely used in computer vision for its efficiency in learning high-resolution features. The hyperparameters were set as follows: *C* = 48, *W* = 20, *L* = 14, and *h* = 6 [20], [40]. The output-layer size was configured to match the number of target parameters for each dataset configuration (see Sections 3.2 and 3.3).

In this section, we first evaluate the performance of the MMgen neural networks for efficient *M*_*avg*_ data generation (Section 3.1). We then compare the performance of the two-step analyzing algorithm trained using the RCWA dataset (Section 3.2) and the *M*_*avg*_ dataset (Section 3.3). Finally, we validate our approach using experimentally measured data (Section 3.4).

### 3.1 Generation of *M*_avg_ using MMgen neural networks

The *M*_avg_ data-generation framework consists of two sequential MMgen neural networks designed for efficient *M*_avg_ computation. The first network generates the MM for a given *p*_sample_ using three inputs: (i) the reference parameter vector *p*_ref_ (4 × 1), (ii) the reference MM, *MM* (*p*_ref_, *A*_ref_) (320 × 15 × 1) obtained from the RCWA-based library, and (iii) the difference (*p*_sample_ − *p*_ref_) (4 × 1). The target output is *MM* (*p*_sample_, *A*_ref_) (320 × 15 × 1), based on the RCWA dataset. The second MMgen network refines this output to generate *MM* (*p*_sample_, *A*_exp_) (320 × 15 × 1) using three inputs: (i) the MM output of the first network (320 × 15 × 1), (ii) *p*_sample_(4 × 1), and (iii) *A*_exp_ (2 × 1) as inputs. This network accounts for variable measurement-angles, with further details of the dataset provided in Supplementary Section 3. Both MMgen networks share a Swin U-Net backbone, which is widely recognized for its efficient learning capabilities in high-resolution image segmentation tasks. The hyperparameters were set as follows: *C* = 48, *W* = 20, *L* = 14, and *h* = 6 [21], [41].

**Figure 5:**
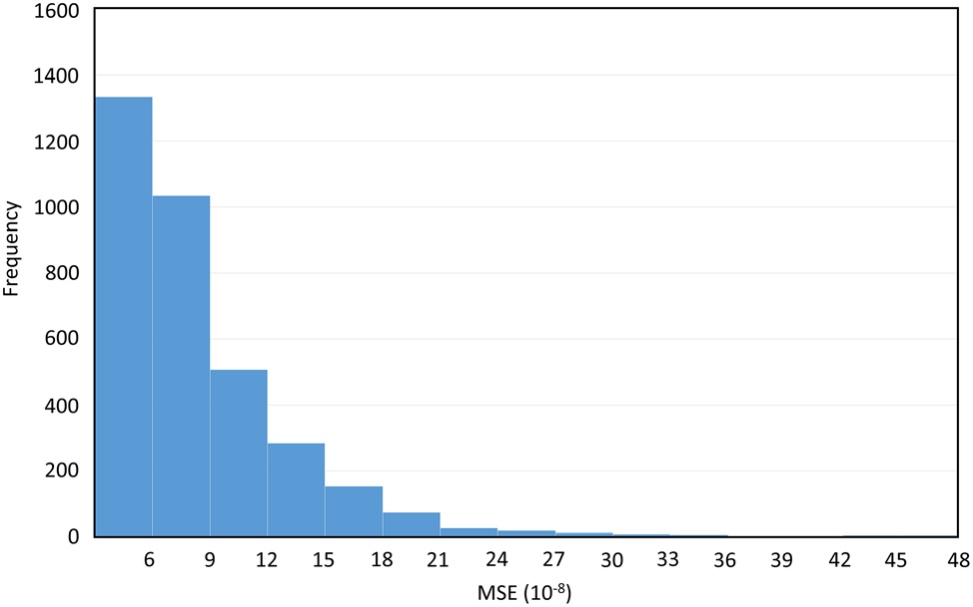
Histogram of MSE values between the MMs generated by the MMgen neural networks and those calculated by RCWA for 3,510 test datasets.

**Figure 6:**
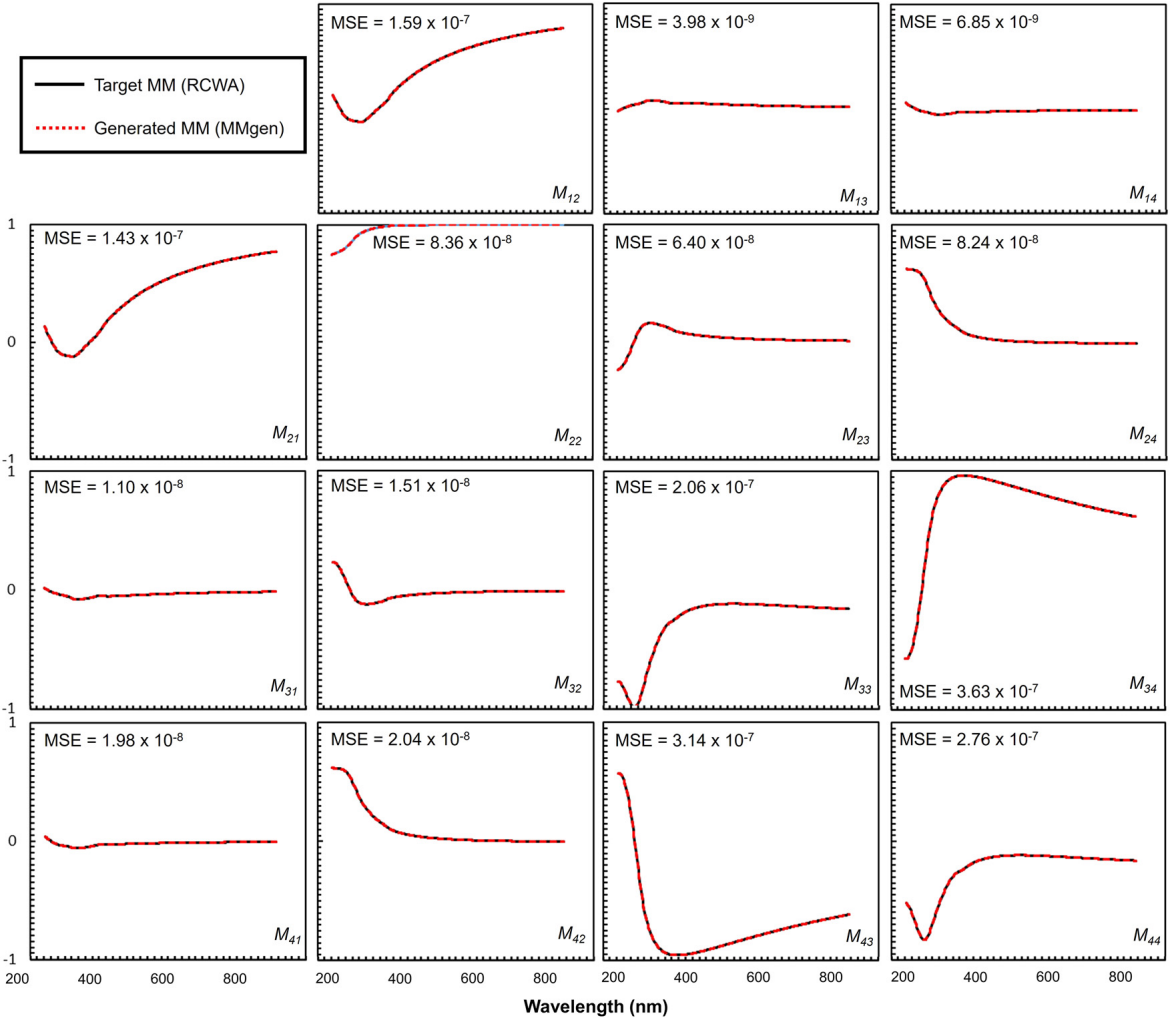
Comparison of MM spectra predicted by the MMgen neural networks with those obtained from RCWA simulations.

After training, the two networks were connected sequentially and evaluated on 3,510 test samples from the RCWA dataset with variable *A*_exp_, achieving an average MSE of 9.99 × 10^−8^. As shown in the histogram of Figure 5, most test samples exhibit MSE values below 9 × 10^−8^. Figure 6 presents an example case with *p*_sample_ = (80.03, 38.61, 9.06, and −7.13 *nm*) and *A*_exp_ = (69.98°, 43.80°), yielding an MSE of 1.1 × 10^−7^, demonstrating high accuracy across the full spectral range and all MM elements. For *M*_avg_ generation, the two networks were executed in batch mode on an NVIDIA A40 GPU (48 GB). With a total model size of ∼82 MB, a batch size of 512 provided the optimal balance between computation time (∼7 s for 10,000 cases), GPU memory usage (∼42 GB), and data transfer latency. Table 1 summarizes inference times and GPU memory usage for different batch sizes.

**Table 1:** GPU memory usage and inference time for different batch sizes during *M*_avg_ generation using the MMgen neural networks for 10,000 samples.

Batch size	GPU memory (MB)	Time per batch (s)	Total time (s)
1	2,496	0.049	492.62
2	2,578	0.049	244.61
4	2,864	0.049	123.84
8	3,196	0.050	62.34
16	3,790	0.050	31.43
32	5,402	0.051	15.99
64	8,542	0.053	8.36
128	13,818	0.093	7.36
256	25,134	0.177	7.11
512	43,904	0.349	7.00

These results confirm that MMgen enables efficient and accurate *M*_avg_ generation across diverse structural and measurement conditions.

### 3.2 Two-step analyzing algorithm trained using an RCWA dataset

The two-step analyzing algorithm was initially trained using only the RCWA dataset, employing supervised learning to predict the four structural parameters. The neural network inputs and targets were defined as follows:

**Step 1**: The input was the MM corresponding to the perfect periodic structure *p*_exp_, and the target was the node *p*_n_ (4 × 1) with the smallest Euclidean distance from *p*_exp_.

**Step 2**: The inputs included the MMs corresponding to the neighboring *p*_n+1_ and *p*_n−1_ nodes (extracted from the RCWA-based library), along with the original MM. The target was the relative displacement vector, *p*_relative_ = *p*_exp_ − *p*_n_ (4 × 1).

### 3.3 Two-step analyzing algorithm trained using a dataset accounting for structural variations and measurement-angle misalignment

To improve prediction accuracy, the two-step analyzing algorithm was trained using a combined dataset comprising the RCWA dataset with variable *A*_exp_ and the *M*_*avg*_ dataset, yielding a total of 70,204 samples. Supervised learning was employed to predict four structural parameters and two measurement angles.

**Step 1**: The input was the *M*_avg_ corresponding to (*p*_exp_[*p*_*c*_, *p*_*r*_], *A*_exp_), and the target was the node *p*_*n*_ (4 × 1) with the smallest Euclidean distance from (*p*_exp_[*p*_*c*_]).

**Step 2**: The inputs included MMs for the +1 order (maximum range/angle) and −1 order (minimum range/angle), together with the original *M*_avg_. The target was the relative displacement vector (6 × 1), (*p*_relative_, *A*_relative_) = (*p*_exp_[*p*_*c*_]−*p*_*n*_, *A*_exp_ −*A*_*n*_), with *A*_*n*_ = (70°, 45°).

**Figure 7:**
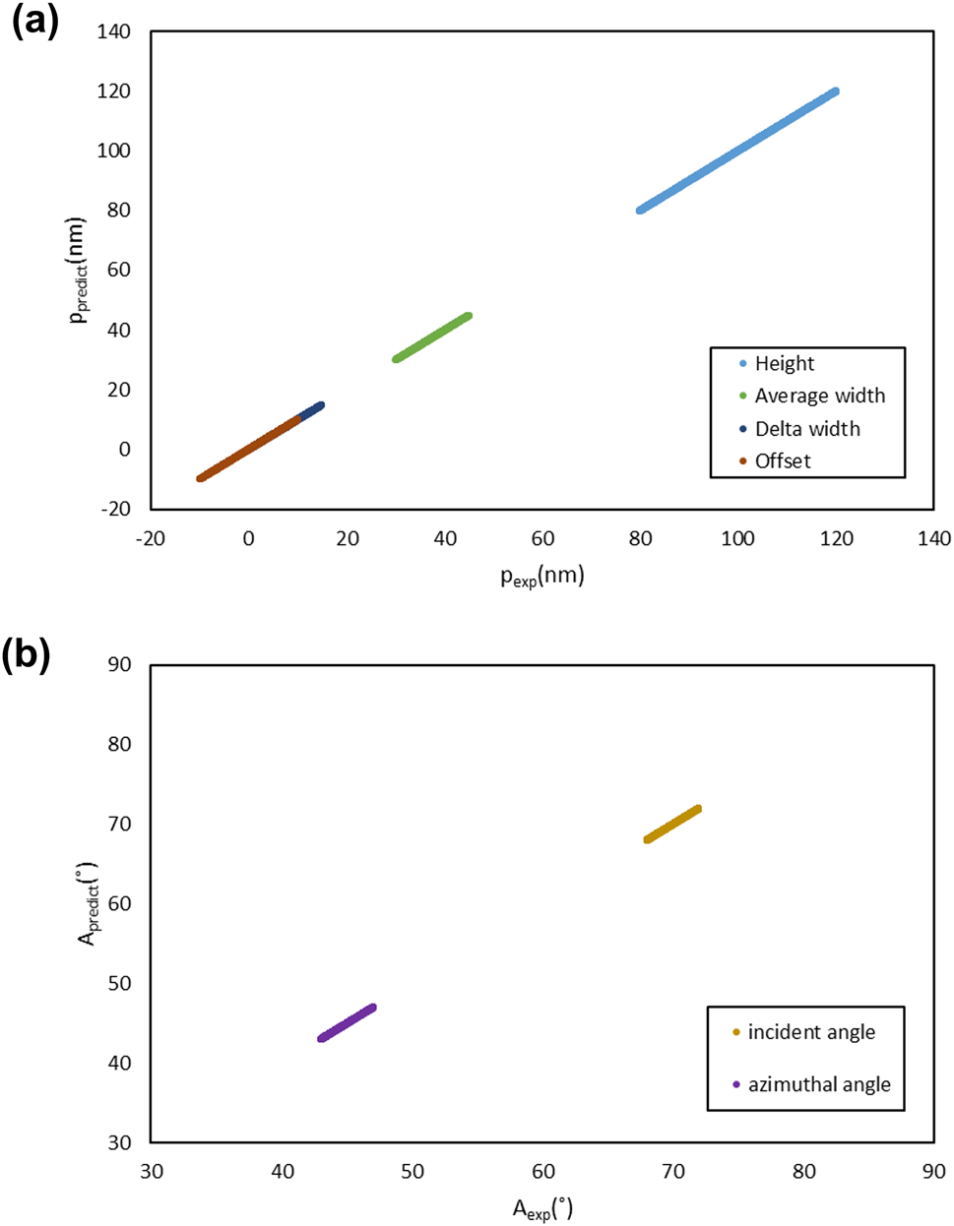
Evaluation of structural parameter (p) and measurement-angle (A) prediction performance on the test datasets: (a) p predictions using the algorithm trained on the *M*_avg_ dataset. (b) A predictions using the algorithm trained using the *M*_avg_ dataset.

Both algorithms were validated using the *M*_avg_ test dataset by evaluating their mean absolute errors (MAEs). As summarized in Table 2, the algorithm trained using the RCWA dataset showed relatively high MAEs for structural parameters, whereas the algorithm trained using the *M*_avg_ dataset achieved consistently low MAEs for both structural parameters and measurement angles. Prediction–target distributions for the algorithm trained on the *M*_avg_ dataset are presented in Figure 7(a) and (b), demonstrating that training with the *M*_avg_ dataset effectively enables the algorithm to account for structural variations of the specimen and measurement-angle misalignment.

**Table 2:** Mean absolute error (MAE) for the prediction of structural parameters and measurement-angle for the *M*_avg_ test set (7,020 samples).

MAE (nm, degree)	Height	Average width	Delta width	Offset	Incident angle	Azimuthal angle
Algorithm trained using the RCWA dataset	5.038	4.910	8.542	0.273	–	–
Algorithm trained using the *M*_avg_ dataset	0.023	0.035	0.044	0.031	0.003	0.011

### 3.4 Experimental evaluation with the fabricated 1D grating

**Figure 8:**
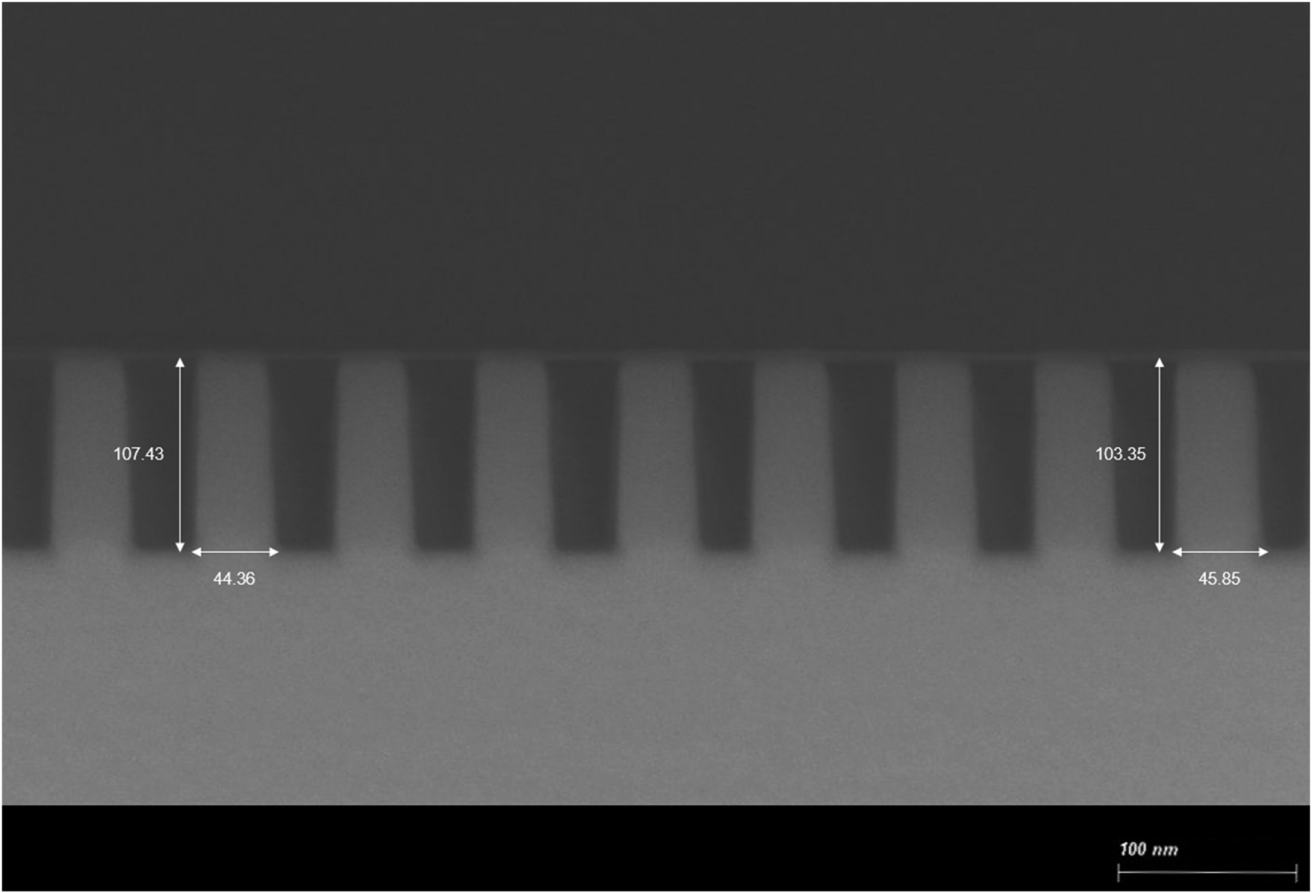
Cross-sectional SEM image of the fabricated 1D grating nanostructure.

To evaluate the performance of the proposed algorithm on real fabricated structures, a one-dimensional SiO_2_ grating with a designed period of 76 nm was fabricated on a silicon substrate, as shown in Figure 8. MMSE measurements (Woollam RC2, J.A. Woollam Co., Nebraska) were conducted at an incident angle of 70° and an azimuthal angle of 45° across 50 points within the patterned region. SEM imaging revealed noticeable linewidth variation across the sample, with representative values of 44.36 nm and 45.85 nm. Mean values and 95 % confidence intervals for each structural parameter were extracted from SEM images obtained at 20 different locations.

Table 3 summarizes the nominal design parameters, SEM-measured values, and average predictions from the two-step algorithms trained using the RCWA dataset and the *M*_avg_ dataset, respectively. Detailed experimental results, including additional parameter configurations, are provided in Supplementary Section 4. The most significant discrepancy occurred in the delta-width parameter. The algorithm trained using the RCWA dataset substantially overestimated this value (16.15 nm), whereas the algorithm trained using the *M*_*avg*_ dataset produced a prediction of 5.39 nm, which closely matched the SEM-measured value of 5.7 ± 1.08 nm.

**Table 3:** Comparison of predicted structural parameters from the two-step algorithms with SEM-measured values for the 1D SiO_2_ grating.

(nm, degree)	Height	Average width	Delta width	Offset	Incident angle	Azimuthal angle
Nominal value	100.00	38.00	0.00	–	–	–
SEM	105.49 ± 0.86	40.93 ± 0.81	5.7 ± 1.08	2.29 ± 0.56	–	–
Predicted value (Algorithm trained using RCWA dataset)	108.05	39.77	16.15	1.67	–	–
Predicted value (Algorithm trained using *M*_avg_ dataset)	105.6	39.86	5.39	2.39	70.08	47.7

Overall, the RCWA-trained model showed noticeable deviations from SEM measurements, while the *M*_*avg*_-trained model – explicitly incorporating fabrication-induced structural variations – exhibited strong agreement with all SEM-derived parameters. Notably, the delta-width prediction error was reduced to only 0.1 nm.

The *M*_*avg*_-dataset trained algorithm also estimated the measurement angles with high fidelity, predicting an incident angle of 70.08° and an azimuthal angle of 47.7°, both consistent with the experimental setup. In summary, experimental validation using MMSE measurements on a real fabricated specimen demonstrates that the algorithm trained with the *M*_*avg*_ dataset achieves the highest overall accuracy. By integrating structural variations and measurement-angle uncertainty into the training process, the algorithm achieved an MAE below 0.4 nm across the four structural parameters and delivered superior performance in predicting delta width. These results highlight its strong capability for precise metrology of fabrication-induced nanoscale structural variations.

## 4 Conclusions

We proposed an enhanced two-step analyzing algorithm for extracting structural parameters from Mueller matrix spectroscopic ellipsometry data, explicitly accounting for fabrication-induced nanoscale structural variations and measurement-angle misalignment. By incorporating these practical nonidealities, the method enables highly accurate analysis under realistic experimental conditions. Central to this approach is the introduction of average Mueller matrix *M*_*avg*_ data, which represent statistical distributions of nanoscale structures within the illuminated region. Using this concept, a new dataset was constructed and used to train the two-step analyzing algorithm through a dedicated *M*_*avg*_ data-generation framework. To support large-scale data preparation, we developed a high-throughput neural network–based MM-generation model (MMgen), which approximates RCWA simulations with significantly reduced computational cost. MMgen achieved a mean squared error of 9.99 × 10^−8^ when validated against RCWA-simulated Mueller matrices, confirming its reliability across a broad range of structural parameters. Experimental validation using a fabricated o SiO_2_ grating further demonstrated the robustness of the proposed framework. The algorithm achieved a dimension prediction error below 0.4 nm when compared with SEM-based measurements, accurately capturing fabrication-induced structural deviations. Overall, the proposed algorithm provides a precise and computationally efficient solution for SE-based metrology, with strong potential for application in semiconductor manufacturing, display technologies, and nanophotonic device characterization.

## Supplementary Material

Supplementary Material Details

## References

[1] Fujiwara H. (2007). *Spectroscopic Ellipsometry: Principles and Applications*.

[2] Aspnes D. E. (2014). Spectroscopic ellipsometry – Past, present, and future. *Thin Solid Films*.

[3] Gostimirovic D., Xu D. X., Liboiron-Ladouceur O., Grinberg Y. (2022). Deep learning-based prediction of fabrication-process-induced structural variations in nanophotonic devices. *Acs Photonics*.

[4] Jiang Z. J., Gan Z. F., Liang C. W., Li W. D. (2024). Generic characterization method for nano-gratings using deep-neural-network-assisted ellipsometry. *Nanophotonics*.

[5] Zollner S. (2013). Spectroscopic ellipsometry for inline process control in the semiconductor industry. *Ellipsometry at the Nanoscale*.

[6] Cattelan D. (2013). Thin film applications in research and industry characterized by spectroscopic ellipsometry. *Ellipsometry at the Nanoscale*.

[7] Aulika I., Paulsone P., Butikova J., Štucere K., Vembris A. (2025). Comprehensive optical characterization of organic thin films for OLED applications via spectroscopic ellipsometry. *Optical Materials: X*.

[8] Oiwake K., Nishigaki Y., Fujimoto S., Maeda S., Fujiwara H. (2021). Fully automated spectroscopic ellipsometry analyses: Application to MoO thin films. *J. Appl. Phys*..

[9] Yoo S., Park Q. H. (2022). Spectroscopic ellipsometry for low-dimensional materials and heterostructures. *Nanophotonics*.

[10] Liu S. (2022). Machine learning aided solution to the inverse problem in optical scatterometry. *Measurement*.

[11] Ichikawa H. (1998). Electromagnetic analysis of diffraction gratings by the finite-difference time-domain method. *J. Opt. Soc. Am. A*.

[12] Polycarpou A. C. (2006). *Introduction to the Finite Element Method in Electromagnetics*.

[13] Pham H. L., Alcaire T., Soulan S., Le Cunff D., Tortai J. H. (2022). Efficient rigorous coupled-wave analysis simulation of mueller matrix ellipsometry of three-dimensional multilayer nanostructures. *Nanomaterials-Basel*.

[14] Hilfiker J. N., Hong N. N., Schoeche S. (2022). Mueller matrix spectroscopic ellipsometry. *Adv. Opt. Technol.*.

[15] Guo C. F. (2023). A combination of library search and Levenberg-Marquardt algorithm in optical scatterometry. *Thin Solid Films*.

[16] Levenberg K. (1944). A method for the solution of certain non-linear problems in least squares. *Quarterly Appl. Math.*.

[17] Gavin H. P. (2019). The Levenberg-Marquardt algorithm for nonlinear least squares curve-fitting problems. *Dep. Civil Environ. Eng. Duke Univer. August*.

[18] Li Y. F., Wu Y. F., Yu H. S., Takeuchi I., Jaramillo R. (2021). Deep learning for rapid analysis of spectroscopic ellipsometry data. *Adv. Photon. Res.*.

[19] Liu J. C., Zhang D., Yu D. Q., Ren M. X., Xu J. J. (2021). Machine learning powered ellipsometry. *Light-Sci. Appl.*.

[20] Jung J. (2023). Geometric analysis algorithm based on a neural network with localized simulation data for nano-grating structure using Mueller matrix spectroscopic ellipsometry. *Opt. Express*.

[21] Jung J. W. (2025). Neural network-based analysis algorithm on Mueller matrix data of spectroscopic ellipsometry for the structure evaluation of nanogratings with various optical constants. *Nanophotonics*.

[22] Chen K. F., Zhao B., Fan S. H. (2018). MESH: A free electromagnetic solver for far-field and near-field radiative heat transfer for layered periodic structures. *Comput. Phys. Commun*..

[23] Politano G. G., Versace C. (2023). Spectroscopic ellipsometry: Advancements, applications and future prospects in optical characterization. *Spectroscopy J.*.

[24] Käseberg T. (2022). Mueller Matrix ellipsometric approach on the imaging of sub-wavelength nanostructures. *Front. Phys.*.

[25] Borna A., Progler C., Blaauw D. (2005). Correlation analysis of CD-variation and circuit performance under multiple sources of variability. *Des. Process Integration Microelectron. Manuf. Iii*.

[26] Lorenz J. K. (2018). Process Variability for Devices at and beyond the 7 nm node. *Ecs J. Solid State Sci.*.

[27] Berriel S. N., Feit C., Keller N., Rudawski N. G., Banerjee P. (2022). Spectroscopic ellipsometry and rigorous coupled wave analysis for real time profile evolution of atomic layer deposited films inside SiO nanotrenches. *J. Vac. Sci. Technol. A*.

[28] Raza S., Hammood M., Jaeger N. A. F., Chrostowski L. (2025). Fabrication-aware inverse design with shape optimization for photonic integrated circuits. *Opt. Lett.*.

[29] Pomplun J., Burger S., Schmidt F., Scholze F., Laubis C., Dersch U. (2008). Metrology of EUV masks by EUV-scatterometry and finite element analysis. *Photomask and Next-Generation Lithography Mask Technology Xv*, Yokohama,.

[30] Orji N. G. (2018). Metrology for the next generation of semiconductor devices (vol 1, pg 532, 2018). *Nat. Electron.*.

[31] Jeong W. K., Kim K. H., Park C., Song D. G., Song M., Seo M. H. (2024). Highly accurate, efficient, and fabrication tolerance-aware nanostructure prediction for high-performance optoelectronic devices. *Sci. Rep.-Uk*.

[32] Boning D. S., El-Henawy S., Zhang Z. X. (2022). Variation-Aware methods and models for silicon photonic design-for-manufacturability. *J. Lightwave Technol*..

[33] Tu H. T. (2021). Method for analyzing the measurement error with respect to azimuth and incident angle for the rotating polarizer analyzer ellipsometer. *Crystals*.

[34] Peng L. H., Tang D. W., Wang J., Chen R., Gao F., Zhou L. P. (2021). Robust incident angle calibration of angle-resolved ellipsometry for thin film measurement. *Appl. Optics*.

[35] Jiang Z., Zhang S., Liu J. M., Li Q., Jiang H., Liu S. Y. (2022). Error analysis for repeatability enhancement of a dual-rotation mueller matrix ellipsometer. *Front Phys.*.

[36] Gu H. G., Liu S. Y., Chen X. G., Zhang C. W. (2015). Calibration of misalignment errors in composite waveplates using Mueller matrix ellipsometry. *Appl. Optics*.

[37] Moharam M. G., Gaylord T. K. (1981). Rigorous coupled-wave analysis of planar-grating diffraction. *J. Opt. Soc. Am.*.

[38] Garcia-Caurel E., De Martino A., Gaston J. P., Yan L. (2013). Application of spectroscopic ellipsometry and mueller ellipsometry to optical characterization. *Appl. Spectrosc*..

[39] Robinson J. C., Sendelbach M. J. (2023). Metrology, inspection, and process control XXXVII. *Proc. SPIE*.

[40] Liu Z. (2021). Swin transformer: Hierarchical vision transformer using shifted windows. *2021 Ieee/Cvf International Conference on Computer Vision (Iccv 2021)*.

[41] Cao H. (2022). Swin-unet: Unet-like pure transformer for medical image segmentation. *European Conference on Computer Vision*.

