# Supplementary material for: AI-based analysis algorithm incorporating nanoscale structural variations and measurement-angle misalignment in spectroscopic ellipsometry
Source: Nanophotonics. 2025 Dec 9;14(27):5621–32. doi: 10.1515/nanoph-2025-0515 (PMC12717921; doi:10.1515/nanoph-2025-0515)
Supplement: Supplementary file 1 — Supplementary Material Details [file j_nanoph-2025-0515_suppl_001.docx]

Juwon Jung, Leeju Hwang, Nagyeong Kim, Kibaek Kim, Seri Kim, Jongkyoon Park, Won Chegal, Yong Jai Cho, and Young-Joo Kim ^[[1]](#footnote-1)^

AI-based Analysis Algorithm Incorporating Nanoscale Structural Variations and Measurement-Angle Misalignment in Spectroscopic Ellipsometry: SUPPLEMENTAL DOCUMENTS

1. **Validation results based on multiple cases using FDTD simulation**

In Section 2.1 of the manuscript, we discussed cases in which structural variations are present. As one example, we examined a structure composed of four consecutive dimensions increasing at equal intervals—(35, 37, 39, 41 nm), corresponding to p_c_ = 38 nm and p_r_ = 6 nm. For this case, we compared the averaged MM with the direct MM calculated using FDTD for a non-uniform structure with geometrical variations, focusing on the *M_23_* element.

In addition to *M_23_*, the spectral comparison across all MM elements can be examined in Fig. S1. The mean squared error (MSE) for each MM element, shown in the corresponding plots, exhibits some variation across elements. However, since none of the elements show significant discrepancies, we confirm that the agreement between the averaged MM and the direct FDTD-calculated MM remains consistently high across all MM elements.


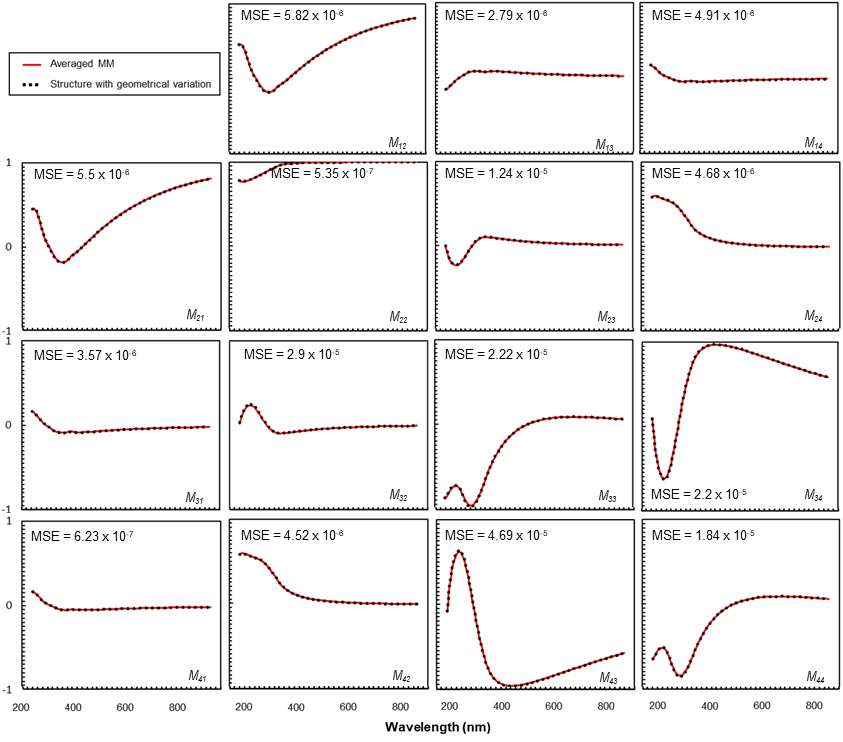


**Fig. S1: Comparison of the averaged MM and direct MM calculated using FDTD for a non-uniform structure with geometrical variations (p_c_ = 38 nm, p_r_ = 6 nm).**

To ensure statistical relevance under feasible conditions, the width parameter was defined from 30 to 46 nm in 1nm increments for total 17 structural cases. Among them, we selected 40 combinations consisting of 4 geometries variations, and for every combination, both the averaged MM and the FDTD-calculated MM were obtained.

As in Fig. 2(c), two representative non-uniform structures composed of four structure among the 40 cases were examined to compare the averaged MM with the FDTD-calculated MM for the *M_23_* element. These two cases—(33, 35, 37, 39 nm), corresponding to p_c_ = 36 nm and p_r_ = 6 nm, and (38, 40, 42, 44 nm), corresponding to p_c_ = 41 nm and p_r_ = 6 nm—are presented in Fig. S2. Both cases show a high degree of agreement between the averaged MM and the direct FDTD results, confirming that the consistency observed in Fig. 2(c) holds for different structural combinations as well.


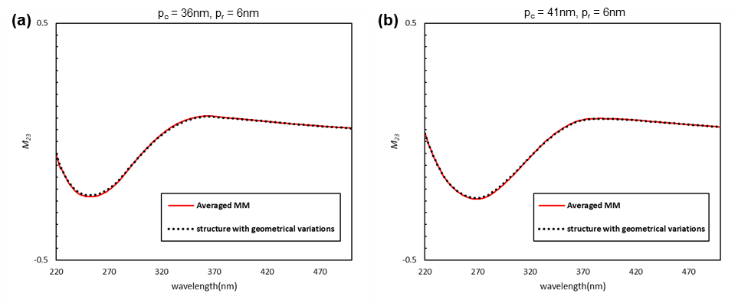


**Fig. S2: Comparison of the M23 element between the averaged MM and the direct MM calculated using FDTD for non-uniform structures with geometrical variations: (a) p_c_ = 36 nm, p_r_ = 6 nm, and (b) p_c_ = 41 nm, p_r_ = 6 nm.**

We additionally examined selected cases among the 40 distribution samples to verify whether the MM remains unique with respect to the defined parameter region.

First, we compared two cases with different p_r_ values while keeping p_c_ identical. For p_c_ = 38 nm with p_r_ = 6 nm and 12 nm (Fig. S3(a)), and for p_c_ = 36 nm with p_r_ = 6 nm and 12 nm (Fig. S3(b)), the MM spectra changed when p_r_ differed, even though p_c_ was the same.

Second, we compared cases with different p_c_ values while keeping p_r_ fixed. When p_c_ = 38 nm and 36 nm with p_r_ = 6 nm (Fig. S3(c)), the MM spectra again differed, despite having identical p_r_.

These observations confirm that the MM exhibits uniqueness with respect to the defined parameter region.


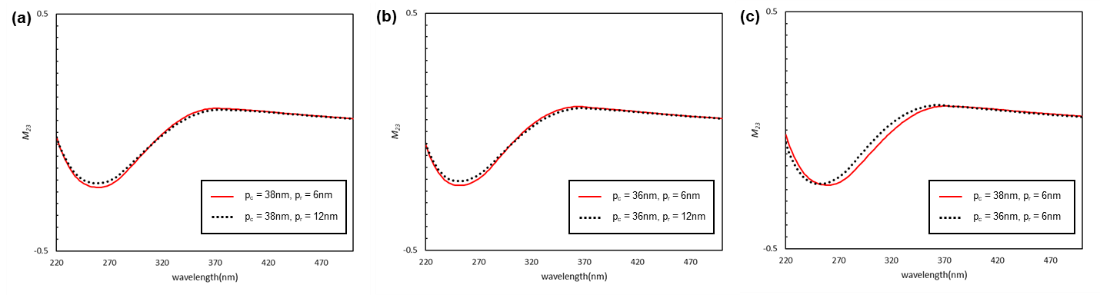


**Fig. S3: Comparison of the *M_32_* element : (a) between p_c_ = 38 nm, p_r_ = 6 nm and p_c_ = 38 nm, p_r_ = 12 nm; (a) between p_c_ = 36 nm, p_r_ = 6 nm and p_c_ = 36 nm, p_r_ = 12 nm; (c) between p_c_ = 38 nm, p_r_ = 6 nm and p_c_ = 36 nm, p_r_ = 6 nm.**

1. **Sampling Strategy for Generation of Averaged Mueller Matrix (*M_avg_*)**

Random Symmetric Sampling (RSS) was employed to efficiently generate representative discrete samples in the four-dimensional parameter space defined by structural variations of specimen. While uniform or random sampling methods are possible, increasing the number of sample count for improving representativeness usually leads the computational cost and time. Thus, it is important to select an efficient sampling strategy for balancing the computational resources with sufficient data representativeness.

The key innovation of RSS lies in exploiting symmetry to efficiently populate the entire parameter space. For every initial $p_{\Delta}$ sample, all $2^{4}=16$ combinations of sign inversion are applied to each component of $p_{\Delta}$, systematically generating all symmetric counterparts:

$$p_{sam}=(h_{c}\pm h_{\Delta},aw_{c}\pm aw_{\Delta},dw_{c}\pm dw_{\Delta},off_{c}\pm off_{\Delta})$$

This procedure ensures that each local random point is expanded to occupy all 16 sub-regions evenly, resulting in a total sample count of:

$$N=16\times n$$

where n represents the number of initial random deviation vectors.

For example, a single random sample $p_{sam,1}$ is expanded into the complete set of 16 sign permutations:

$$p_{sam,1}=(h_{c}+h_{\Delta},aw_{c}+aw_{\Delta},dw_{c}+dw_{\Delta},off_{c}+off_{\Delta})$$

$$p_{sam,2}=(h_{c}+h_{\Delta},aw_{c}+aw_{\Delta},dw_{c}+dw_{\Delta},off_{c}-off_{\Delta})$$

$$\vdots$$

$$p_{sam,16}=(h_{c}-h_{\Delta},aw_{c}-aw_{\Delta},dw_{c}-dw_{\Delta},off_{c}-off_{\Delta})$$

The advantage of this approach becomes evident when determining the optimal sample size. We systematically determined the minimal sample count required to achieve acceptable accuracy by selecting the minimal N such that the mean squared error (MSE) between the MM of the perfect periodic structure corresponding to p_c_ and *M_avg_* realizations falls within a sufficiently converged region.

We compared our proposed RSS method with a conventional grid-based uniform sampling approach to evaluate efficiency, as shown in Fig. S4. RSS achieves an MSE below 3.85 × 10^-7^ with as few as 64 samples, whereas the grid-based uniform sampling method requires more than 50,000 samples to reach a comparable accuracy level. This demonstrates the significantly higher efficiency and accuracy of RSS over conventional uniform sampling strategies. This represents more than two orders of magnitude improvement in computational efficiency. The rapid convergence of RSS stems from its systematic coverage of the parameter space through symmetric expansion, ensuring that fabrication-induced structure variations can be captured more effectively than grid-based uniform sampling.

By focusing initial sampling within a single sector of the parameter space and subsequently applying symmetric expansion about the center, this method substantially reduces the required number of distinct initial random samples while preserving comprehensive spatial coverage. Unlike the grid-based uniform sampling, which treats all parameter combinations equally regardless of their physical relevance, RSS focuses computational effort on parameter combinations that are more likely to occur in realistic fabrication scenarios.

To ensure consistent sampling density across different parameter ranges, the total number of samples is determined by multiplying the sampling density with the four-dimensional parameter space volume. The parameter space volume is calculated as the product of all four individual parameter ranges (height range times average width range times delta width range times offset range). The sampling density of 96 points per unit hyper-volume of one cubic nanometer serves as the multiplication factor.


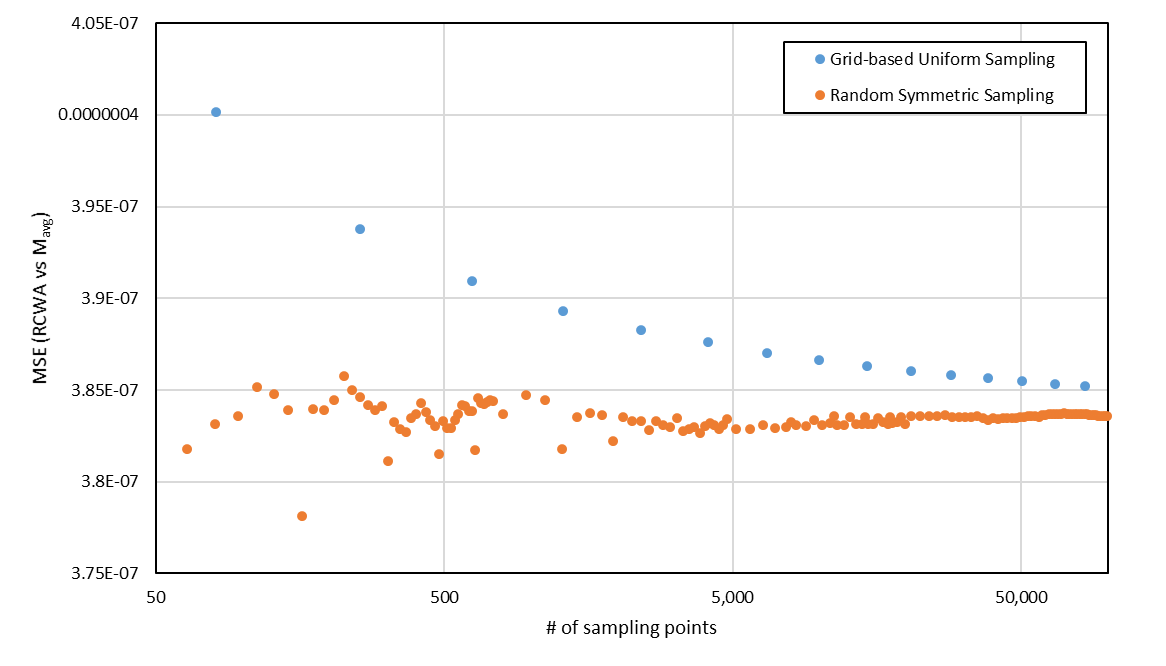


**Fig. S4: Comparison of MSE convergence between Grid-based Uniform Sampling and Random Symmetric Sampling.**

1. **Dataset details for algorithms**

In Section 3, to examine the effectiveness of newly proposed algorithm for structural variations and measurement‑angle misalignment, the performance evaluation was first conducted with the MMgen neural networks (Section 3.1). Then two separate evaluation processes are followed for the comparison, by the two-step analyzing algorithm trained using the RCWA dataset (Section 3.2) and the two-step analyzing algorithm trained using the *M_avg_* dataset (Section 3.3). This supplementary section clarifies the resources used by these three algorithms, including reference libraries and separate training/evaluation datasets. Finally, they are summarized in Table S1.

**Datasets and library for Section 3.1 (MMgen neural networks)**

MMgen neural networks comprises two sequential neural networks that generate MM rapidly and accurately for a given parameter combination while referencing an **RCWA-based library**. The RCWA -based library involves only the four structural parameters with the samples at 5nm intervals in each parameter range, yielding 720 MM entries. The first MMgen neural network is trained using the **RCWA dataset** with fixed incident and azimuthal angles at 70° and 45° (35,102 samples) to predict *MM* (p_sample_, A_ref_). The second MMgen neural network is trained using the **RCWA dataset with variable A_exp_** (35,102 samples) to predict *MM* (p_sample_, A_exp_). All MMs generated at each sample point are averaged to form *M_avg_*, which is later used to train the two-step analyzing algorithm in Section 3.3.

**Datasets and library for Section 3.2 (two-step analyzing algorithm trained using the RCWA dataset)**

This algorithm uses the RCWA-based library and the RCWA dataset. The **RCWA-based library** means same library introduced in Section 3.1 (four structural parameters, 5 nm node spacing, 720 MM entries), while the **RCWA dataset** means the training/evaluation dataset prepared at a fixed incident/azimuthal angles (35,102 samples). This configuration excludes the structure variations and measurement-angle uncertainty in the inputs so that results provide a clear baseline for comparison with the algorithm trained using the *M_avg_* dataset in Section 3.3.

**Datasets and library for Section 3.3 (two-step analyzing algorithm trained using the *M_avg_* dataset)**

This algorithm uses the ***M_avg_* -based library** and the ***M_avg_* dataset**. The ***M_avg_* -based library** pairs node points sampled at 5 nm intervals with distinct angle and structural-range settings (maximum: 72°, 49°, 10 nm; minimum: 68°, 41°, 0 nm), yielding 1,440 MM entries. The ***M_avg_* dataset** is the training/evaluation dataset that represents each of the four parameters with a center value and a range together with the two measurement-angles; it is constructed by combining *M_avg_* generated via the MMgen neural networks (35,102 samples) with the **RCWA dataset with variable A_exp_** (equivalent to p_r_ = 0; 35,102 samples), totaling 70,204 samples.

**Common settings**

All resources follow the parameter ranges defined in Section 2.1; the spectral range is 220–858 nm at 2 nm intervals (320 wavelength points); each MM input uses 15 components (excluding *M_11_*). All training/evaluation datasets are randomly split into training, validation, and test sets with a ratio of 8:1:1. In both Sections 3.2 and 3.3, the two-step network architecture is identical across experiments: with input size of 320×15×1 for Step 1, input size of 320×15×3 (original MM plus ±1‑order nodes) for Step 2, and Swin Transformer hidden layers configured as C = 48, W = 20, L = 14, and h = 6. Table S1 summarizes the dataset and library used to the each corresponding algorithm and reports the number of sample count.

**Table. S1: Datasets and libraries used for MMgen and two-step analyzing algorithms.**

| **Algorithm** | **Dataset** | **# of total data** | **Measurement-angle**  **variable** | **with structural**  **variation** |
| --- | --- | --- | --- | --- |
| **MMgen neural networks** | **RCWA-based library** | 720 | X | X |
|  | **RCWA dataset** | 35,102 | X | X |
|  | **RCWA dataset**  **with variable A_exp_** | 35,102 | O | X |
| **2-step analyzing algorithm trained using RCWA dataset** | **RCWA-based library** | 720 | X | X |
|  | **RCWA dataset** | 35,102 | X | X |
| **2-step analyzing algorithm trained using *M_avg_* dataset** | ***M_avg_* –based library** | 1,440 | O | O |
|  | ***M_avg_* dataset** | 70,204 | O | O |

1. **Comparison of Individual Algorithms Incorporating Structural Variation and measurement-angle**

To further clarify the contribution of structural variation incorporated in the proposed analysis framework, we conducted an additional experiment using a supplementary algorithm configuration that incorporates only the structural variation parameter Ω (variable: p, Ω). This configuration was evaluated alongside the baseline model using only the structural parameters (variable: p) and the proposed full model (variable: p, Ω, A), which integrates both structural variation and angle-related uncertainty.

All prediction results were compared against the average SEM-measured structural parameters of the fabricated specimen, and the outcomes are summarized in Table S2. When only structural variation (Ω) was included, the height prediction error decreased substantially, and the delta width error also improved relative to the baseline; however, the average width error increased.

Finally, the proposed model achieved consistent accuracy improvements across height, average width, delta width and offset after applying both Ω and A. This confirms that while Ω alone contributes meaningful correction, the combination of structural variation and angle uncertainty is essential for reliably extracting structural parameters from fabricated nanostructures.

**Table. S2: Comparison on the predicted values with SEM measured values for the 1D SiO_2_ grating.**

| **(nm, degree)** | **Height** | **Average width** | **Delta width** | **Offset** | **Incident angle** | **Azimuthal angle** |
| --- | --- | --- | --- | --- | --- | --- |
| **SEM** | 104.95 ± 0.64 | 39.7 ± 0.71 | 5.5 ± 0.77 | 2.29 ± 0.56 |  |  |
| **Predicted value (variable : p)** | 108.05 | 39.77 | 16.15 | 1.67 |  |  |
| **Predicted value (variable : p, Ω)** | 104.73 | 42.92 | 9.26 | 1.88 |  |  |
| **Predicted value (variable : p, Ω, A)** | 105.60 | 39.86 | 5.39 | 2.39 | 70.08 | 47.7 |

1. ^+^Juwon Jung and Leeju Hwang contributed equally to this work as shared first authors.

   **Corresponding author:** **Young-Joo Kim**, Department of Mechanical Engineering, Yonsei University, Seoul, Republic of Korea; yjkim40@yonsei.ac.kr; https://orcid.org/0000-0002-5378-422X

   **Juwon Jung,** **Leeju Hwang, Nagyeong Kim, Kibaek Kim and Seri Kim**, Department of Mechanical Engineering, Yonsei University, Seoul, Republic of Korea;

   **Jongkyoon Park, Won chegal and Yong Jai Cho:** Semiconductor Integrated Metrology Team, Korea Research Institute of Standards and Science, Daejeon, Republic of Korea; [↑](#footnote-ref-1)
